# Reactogenicity and Immunogenicity Against MPXV of the Intradermal Administration of Modified Vaccinia Ankara Compared to the Standard Subcutaneous Route

**DOI:** 10.3390/vaccines13010032

**Published:** 2024-12-31

**Authors:** Valentina Mazzotta, Pierluca Piselli, Alessandro Cozzi Lepri, Giulia Matusali, Eleonora Cimini, Rozenn Esvan, Francesca Colavita, Roberta Gagliardini, Stefania Notari, Alessandra Oliva, Silvia Meschi, Rita Casetti, Giulia Micheli, Licia Bordi, Alessandro Giacinta, Germana Grassi, Saba Gebremeskel Tekle, Claudia Cimaglia, Jessica Paulicelli, Alessandro Caioli, Paola Gallì, Giulia Del Duca, Miriam Lichtner, Loredana Sarmati, Enrica Tamburrini, Claudio Mastroianni, Alessandra Latini, Paolo Faccendini, Carla Fontana, Emanuele Nicastri, Andrea Siddu, Alessandra Barca, Francesco Vaia, Enrico Girardi, Fabrizio Maggi, Andrea Antinori

**Affiliations:** 1 Clinical Infectious Diseases Department, National Institute for Infectious Diseases Lazzaro Spallanzani IRCCS, 00149 Rome, Italy; valentina.mazzotta@inmi.it (V.M.); rozenn.esvan@inmi.it (R.E.); roberta.gagliardini@inmi.it (R.G.); alessandra.oliva@inmi.it (A.O.); giulia.micheli@inmi.it (G.M.); alessandro.giacinta@inmi.it (A.G.); saba.gebremeskeltekle@inmi.it (S.G.T.); jessica.paulicelli@inmi.it (J.P.); giulia.delduca@inmi.it (G.D.D.); emanuele.nicastri@inmi.it (E.N.); andrea.antinori@inmi.it (A.A.); 2 Clinical Epidemiology Unit, National Institute for Infectious Diseases Lazzaro Spallanzani IRCCS, 00149 Rome, Italy; claudia.cimaglia@inmi.it (C.C.); alessandro.caioli@inmi.it (A.C.); 3 Centre for Clinical Research, Epidemiology, Modelling and Evaluation (CREME), Institute for Global Health, University College London (UCL), London NW3 2PF, UK; a.cozzi-lepri@ucl.ac.uk; 4 Laboratory of Virology, National Institute for Infectious Diseases Lazzaro Spallanzani IRCCS, 00149 Rome, Italy; giulia.matusali@inmi.it (G.M.); francesca.colavita@inmi.it (F.C.); silvia.meschi@inmi.it (S.M.); licia.bordi@inmi.it (L.B.); fabrizio.maggi@inmi.it (F.M.); 5 Cellular Immunology and Pharmacology Laboratory, National Institute for Infectious Diseases, Lazzaro Spallanzani IRCCS, 00149 Rome, Italy; eleonora.cimini@inmi.it (E.C.); stefania.notari@inmi.it (S.N.); rita.casetti@inmi.it (R.C.); germana.grassi@inmi.it (G.G.); 6 Health Direction, National Institute for Infectious Diseases Lazzaro Spallanzani IRCCS, 00149 Rome, Italy; paola.galli@inmi.it; 7 Infectious Diseases Unit, NESMOS Department, Santa Maria Goretti Hospital of Latina, Sapienza University of Rome, 04100 Latina, Italy; miriam.lichtner@uniroma1.it; 8 Infectious Diseases Unit, Tor Vergata University Hospital, 00133 Rome, Italy; sarmati@med.uniroma2.it; 9 Department of Safety and Bioethics, Catholic University of the Sacred Heart, 00136 Rome, Italy; enrica.tamburrini@policlinicogemelli.it; 10 Infectious Diseases Unit, Fondazione Policlinico Universitario A. Gemelli IRCCS, 00136 Rome, Italy; 11 Department of Public Health and Infectious Diseases, Sapienza University of Rome, 00161 Rome, Italy; claudio.mastroianni@uniroma1.it; 12 STI/HIV Unit, San Gallicano Dermatological Institute IRCCS, 00144 Rome, Italy; alessandra.latini@ifo.it; 13 Pharmacy Unit, National Institute for Infectious Diseases Lazzaro Spallanzani IRCCS, 00149 Rome, Italy; paolo.faccendini@inmi.it; 14 Laboratory of Microbiology and Biological Bank Unit, National Institute for Infectious Diseases Lazzaro Spallanzani IRCCS, 00149 Rome, Italy; carla.fontana@inmi.it; 15 General Directorate of Prevention, Ministry of Health, 00144 Rome, Italy; a.siddu@sanita.it (A.S.); segr.dgprev@sanita.it (F.V.); 16 Unit of Health Promotion and Prevention, Directorate of Health and Integration, Lazio Region, 00145 Rome, Italy; abarca@regione.lazio.it; 17 Scientific Direction, National Institute for Infectious Diseases Lazzaro Spallanzani IRCCS, 00149 Rome, Italy; enrico.girardi@inmi.it

**Keywords:** mpox, immunogenicity, reactogenicity, cellular response, humoral response, vaccine, MVA-BN, trial emulation

## Abstract

Background: The recent resurgence of mpox in central Africa has been declared a new public health emergency of international concern (PHEIC) requiring coordinated international responses. Vaccination is a priority to expand protection and enhance control strategies, but the vaccine’s need exceeds the currently available doses. Intradermal (ID) administration of one-fifth of the standard modified vaccinia Ankara (MVA-BN) dose was temporarily authorized during the 2022 PHEIC. Studies conducted before 2022 provided evidence about the humoral response against the vaccinia virus (VACV) after vaccination but not against the mpox virus (MPXV). Moreover, no data are available on the T-cell response elicited by MVA-BN administered subcutaneously or intradermally. Methods: We compare the two vaccine administration routes according to reactogenicity (*n* = 943) and immunogenicity (*n* = 225) of vaccine recipients attending INMI Spallanzani hospital during the 2022 vaccination campaign in Rome, Italy. Results: We found that the ID route elicited higher titers of MPXV-specific IgG (mean difference of 0.26 log_2_, *p* = 0.05) and nAbs (0.24 log_2_, *p* = 0.08) than the subcutaneous (SC) route one month after the complete vaccination cycle. At the same time, no evidence for a difference in cellular response was found. Conclusions: MVA-BN was globally well tolerated despite higher reactogenicity for the ID than the SC route, especially for the reactions at the local injection site. The ID dose-sparing strategy was proven safe and immunogenic and would make vaccination available to more people. Our data support the current WHO recommendation of using the ID route in low–medium-income countries (LMIC), although response data in people infected with the new 1b clade are urgently needed.

## 1. Introduction

Starting in May 2022, an unexpected mpox epidemic caused by the clade IIb spread to 122 countries, with a global case count higher than 99,000 [1]. The rapid increase in confirmed cases led to the subsequent declaration of mpox as a public health emergency of international concern (PHEIC) by the World Health Organization (WHO) [2]. Therefore, the WHO recommended vaccination for high-risk people and other mitigation strategies to control the epidemic [3].

Licensed vaccines against mpox consist of two doses of the third-generation replication-deficient modified vaccinia Ankara produced by Bavarian Nordic (MVA-BN) [4,5]. The standard route of administration of MVA-BN is subcutaneous (SC), with two doses delivered at least 28 days apart. Numerous studies have shown that for other types of vaccines (e.g., rabies, hepatitis B, or influenza vaccines), in both animal and human models, ID immunization results in similar or even enhanced immunogenicity using a fractional dose, despite higher local reactogenicity from the ID than the SC route [6,7].

The safety and immunogenicity of the intradermal (ID) administration of one-fifth of the standard subcutaneous (SC) formulation (dose and route) of MVA-BN has been assessed in humans through a randomized clinical trial (NCT number: NCT00914732) [8]. Although the proportion of local reactions was significantly higher after ID inoculation than that seen with SC, the ID route was considered non-inferior to the SC route regarding neutralization against vaccinia virus, and it was purposed as a valid alternative in the event of an emergency requiring the availability of more doses [9].

In the summer of 2022, it became necessary to rapidly vaccinate as many high-risk people as possible to contain the epidemic [10]. Therefore, due to the limited availability of MVA-BN worldwide, the European Medicine Agency and Food and Drug Administration authorized using the ID route of administration with a reduced dose to extend vaccination [11,12].

Data from large-scale administration of MVA-BN published by Bavarian Nordic confirmed the overall safety of the vaccine with an increased frequency of syncopal events after ID administration [13].

Recently, Frey et al. reported data from a phase 2 open-label trial comparing two 2-dose ID regimens of MVA-BN (one-tenth and one-fifth of SC, respectively) with the standard dose SC regimen. MVA-BN administrated ID at fractional doses was safe, and the vaccination with one-fifth (but not one-tenth) dose demonstrated non-inferior immunogenicity by vaccinia virus (Western Reserve strain) plaque reduction neutralization test (PRNT) after 43 days [14]. However, even though little evidence for a difference in clinical effectiveness between the two different vaccination schedules against mpox was reported from 2022 real-world data [15], comparative data on the immunogenicity of ID route compared to SC are lacking, and those available are not informative regarding the specific humoral response against mpox, nor on T-cell response.

Although the massive vaccination campaigns during the MPXV clade IIb outbreak has helped to control the epidemic in high-income countries [16], outbreaks have constantly been observed in endemic areas over the last year, especially in South Africa, where clade IIb of mpox virus (MPXV) was still detected [17], and in the Democratic Republic of Congo, where clade I acquired the capability of human-to-human transmission through the sexual route [18]. This “new sexually transmitted clade”, named clade Ib, has spread in some neighboring countries where mpox cases have never been reported before (e.g., Burundi, Rwanda, Uganda) [19] and imported cases have also been detected in Europe and other countries around the world (Sweden, Thailand, Germany, India, and the United Kingdom) [20]. This new clade, is particularly worrisome because of its lethality (nearly 3%) [21], leading the WHO to renew the declaration of mpox as a PHEIC on the 14 August 2024 [19].

Vaccination is recommended as one of the main tools to control this new increase in mpox cases. The WHO and the African Centers for Disease Control and Prevention (CDC) are producing efforts to step up the vaccination campaign in low-income countries with limited access. Although it has been estimated by the African CDC that 10 million doses will be needed to contain the current outbreak, the negotiations with Bavarian Nordic aim to obtain around 200,000 doses of MVA-BN [22]. In this setting, enhancing knowledge about the reactogenicity and immunogenicity against MPXV of the ID-sparing strategy compared to the standard SC in people at high-risk of acquiring mpox in a real-world setting is crucial to address the efficient and cost-effective implementation of the arising mpox vaccination campaign.

Here, we report the results of an analysis of observational data comparing the safety, reactogenicity, and immunogenicity (neutralization and T-cell response) specifically directed at the MPXV target between the two vaccination schedules of MVA-BN (Jynneos) delivered during the mpox outbreak and consequent vaccination campaign in Italy over 2022–2023.

## 2. Materials and Methods

### 2.1. Patients’ Enrolment

In the Lazio Region of Italy, the mpox vaccination campaign started on 8 August 2022, and took place in a hospital setting at the Lazzaro Spallanzani National Institute for Infectious Diseases in Rome, which was identified as the only vaccination center in the entire region.

According to the recommendations of the Ministry of Health [23], MVA-BN was administered as pre-exposure prophylaxis to a target population, including laboratory personnel with possible direct exposure to orthopoxviruses (OPXV) and high-risk gay–bisexual men who have sex with men (GBMSM), defined as individuals reporting a recent history of sexually transmitted infections (STIs), multiple sexual partners or participation in group sex events, sexual encounters in clubs/cruising/saunas, or sexual acts associated with the use of Chemical drugs (chemsex). For individuals who had never received the smallpox vaccine (vaccine-naïve or non-primed), the vaccination schedule consisted of a two-dose cycle with a 28-day interval between each dose, while individuals who had received the smallpox vaccine in the past (vaccine-experienced or primed) were administered a single-dose cycle. Of note, in Italy, the smallpox vaccination campaign was stopped in 1977 and officially abrogated in 1981 [24].

The MVA-BN first dose was administered subcutaneously (0.5 mL containing no less than 5 × 10^7^ infectious units) during the first two weeks of the vaccination campaign, after which the intradermal route containing one fifth of volume (0.1 mL) was adopted, following the ministerial indications [25].

For the same reason, the vaccine was administered exclusively using the ID route in those receiving a second dose. A prospective observational cohort was integrated into the framework of this vaccination campaign. Not all patients included in this analysis had available data for all the outcome parameters studied. The majority contributed only a diary with recorded adverse events data, a minority only immunogenicity data, and some contributed to both. Reasons for missing data outcomes included convenience, costs, and laboratory failure results.

### 2.2. Study Protocol

The protocol for the study named Mpox-Vac (“Studio prospettico osservazionale per monitorare aspetti relativi alla sicurezza, all’efficacia, all’immunogenicità e all’accettabilità della vaccinazione anti Monkeypox con vaccino MVA-BN (JYNNEOS) in persone ad alto rischio”) was approved by the INMI Lazzaro Spallanzani Ethical Committee (approval number 41z, Register of Non-Covid Trials 2022). The study protocol was previously described in detail elsewhere [26].

Briefly, all subjects eligible for mpox vaccination according to the ministerial guidelines and who signed a written informed consent form were enrolled in this study. Data measured on laboratory personnel were excluded from the analysis. At baseline (when receiving the first MVA-BN dose, T1), subjects were evaluated for demographic and behavioral characteristics linked to mpox exposure. Information regarding HIV status, CD4 count, HIV pre-exposure prophylaxis (PrEP) uptake, and any history of previous STIs was collected. In non-primed participants, other time points were scheduled at the administration of the second dose (T2) and one month after the completion of the cycle (T3). For the vaccine-experienced individuals (primed) who received a single-dose schedule as a complete vaccination cycle, T2 was the time point corresponding to one month after vaccination completion.

### 2.3. Assessment of Adverse Reactions

As part of the protocol, participants were delivered a paper symptom diary after each vaccine dose (T1 and T2) to collect self-reported adverse effects following immunization (AEFIs) for 28 consecutive days (the 28 days following T1 and the 28 days following T2). Participants returned the completed diaries at their next appointment. Because the SC route was never used as second dose, safety and reactogenicity by administration route could only be compared 28 days after T1. Those who agreed to collect their diary were included in the Group 1 sub-group analysis.

Participants were able to report the presence of systemic symptoms (S-AEFIs) classified as fatigue, muscle pain, headache, gastrointestinal effects, and chills, and local injection site symptoms (LIS-AEFIs), such as redness, induration, and pain. AEFIs were graded by the vaccinees as absent (grade 0), mild (1), moderate (2), or severe (3). Here, we report the results regarding erythema and induration as recalculated with the use of the current FDA Toxicity Grading Scale for Healthy Adult and Adolescent Volunteers Enrolled in Preventive Vaccine Clinical Trials. According to that scale, a diameter of 25 to 50 mm indicates a mild reaction, 51 to 100 mm is a moderate reaction, and more than 100 mm is a severe reaction [27].

### 2.4. Assessment of Immunogenicity

In a specific population of participants for whom blood samples collected at each time point were available, the assessment of the early humoral and cellular immune response was performed (Group 2). Blood samples were collected from eligible participants who gave a specific consent to blood collection at T1 and T2, independently to the participation to the report of the presence of adverse events above described.

#### 2.4.1. MPXV-Specific IgG and Neutralization Assays

Specific anti-MPXV immune response was evaluated by measuring MPXV-specific IgGs and neutralizing antibodies in the serum as previously described [28]. The presence of anti-MPXV IgGs was assessed on immunofluorescence slides in-house prepared with Vero E6 cells (ATCC) infected with an MPXV isolated from the skin lesion of a patient infected with MPXV during the 2022 outbreak (GenBank ON745215.1, clinical sample). Serum samples were tested with a starting dilution of 1:20, and serial two-fold dilutions were performed to determine anti-MPXV IgG titer. MPXV-specific neutralizing antibodies (nAbs) were measured by 50% plaque reduction neutralization test (PRNT_50_) with a starting dilution of 1:10. Specifically, serum samples were heat-inactivated at 56 °C for 30 min and titrated in duplicate in 4 four-fold serial dilutions. Each serum dilution was added to the same volume (1:1) of a solution containing 100 TCID_50_ MPXV isolate (GenBank ON745215.1, clinical sample) and incubated at 37 °C for 2 h. Subconfluent Vero E6 cells were infected with virus/serum mixtures and incubated at 37 °C and 5% CO_2_. After 5 days, the supernatant was carefully discarded, and a crystal violet solution (Diapath S.P.A., Bergamo, Italy) containing 10% formaldehyde (Sigma-Aldrich) was added for 30 min; then, cells were washed with phosphate-buffered saline (PBS, 1×; Sigma-Aldrich, Darmstadt, Germany). Using the Biotek Cytation 5 reader (Agilent, Santa Clara, CA, USA), the number of plaques was counted. The neutralizing titers were estimated by measuring the plaques number reduction as compared to the control virus wells. The highest serum dilution showing at least 50% of the plaque number reduction was indicated as the 50% neutralization titer (PRNT_50_). Each test included serum control (1:10 dilution of each sample tested without virus), cell control (Vero E6 cells alone), and virus control (100 TCID_50_ MPXV in octuplicate).

#### 2.4.2. PBMC Isolation

Using Ficoll density gradient centrifugation (Pancoll human, PAN Biotech, Aldenbach, Germany), peripheral blood mononuclear cells (PBMCs) were isolated, frozen in FBS (Fetal Bovine Serum, Gibco, Grand Island, NY, USA) added with 10% of DMSO (Merck Life sciences, Milan, Italy) at vapors of liquid nitrogen for further experiments.

#### 2.4.3. ELISpot Assay

The frequency of T-cell-specific responses to the MVA-BN vaccine was assessed by Interferon-γ ELISpot assay. Briefly, PBMC were thawed and suspended in a complete medium [RPMI-1640 added of 10% FBS, 1% L-glutamine, and 1% penicillin/streptomycin (Euroclone S.p.A, Pero (MI), Italy)]. Live PBMC were counted by Trypan blue exclusion, plated at 3 × 10^5^ cells per well in ELISpot plates (Human IFN-γ ELISpot plus kit; Mabtech, Nacka Strand, Sweden), and stimulated for 20 h with MOI 1 of the MVA-BN vaccine suspension [JYNNEOS (Smallpox and Monkeypox Vaccine, Live, non-replicating)] and anti-CD28/anti-CD49d (1 μg/mL, BD Biosciences, Franklin Lakes, NJ, USA) at 37 °C (5% CO_2_), using T-cell superantigen (SEB 200 nM, Sigma-Aldrich, Darmstadt, Germany) as positive control. At the end of incubation, the ELISpot assay was developed following the manufacturer’s instructions. The results are expressed as spot-forming cells per 10^6^ PBMCs (SFC/10^6^ PBMCs) in stimulating cultures after subtracting the background (unstimulated culture).

### 2.5. Statistical Analysis

Demographic and epidemiological characteristics of the patients are presented both for the overall sample population and after stratification by route of administration. Continuous variables were described using median and interquartile range (IQR), while categorical variables were summarized using absolute numbers and percentages.

The main characteristics of participants according to the route of administration were compared using the chi-square test or Fisher exact test when appropriate for qualitative variables and the Mann–Whitney or Kruskal–Wallis test for numerical variables.

In the population of vaccinated subjects for whom stored samples were available, we described the raw immunogenic response data by plotting the geometric mean titers (GMT) or SFC at T1 and T2 and the average change over T1-T2 stratified by inoculation strategy received under the natural course. The crude mean T1-T2 change was compared by using the non-parametric Mann–Whitney test for unpaired data.

Then, to compare the overall increase in the average levels (on a log_2_ scale) of IgG and nAb as well as ELISpot response 1 month after the complete vaccination cycle (a single dose for the primed but two doses for the non-primed participants) according to route of administration, we used a marginal model and calculated the average treatment effect (ATE). We used a doubly robust method (using augmented inverse probability weighting-AIPW) to obtain estimates that are robust against misspecification of either the propensity model or the outcome model. Both the propensity and outcome models included HIV and age as confounding variables. Because of the collinearity between being primed and age, we did not further control for the imbalance in proportion of participants who had been primed. For the outcome model, we also used the saturated model, including the interaction parameter between exposure and HIV status with similar results.

The route of administration strategy was classified according to the type of the inoculation used as first dose—as none of the non-primed used SC route at second dose. In other words, in the main analysis, the exact strategies being compared in this emulation analysis were a homologous (ID+ID) vs. heterologous (SC+ID) complete course of vaccination. We also hypothesized that HIV might be an effect measure modifier for the causal effect of interest. We therefore performed a formal interaction test by including an additional parameter in the regression model to verify this hypothesis. Finally, we also restricted the analysis to only primed participants to compare the responses to a single dose of SC vs. a single dose of ID.

We also used the collected diaries to calculate the prevalence of reported AEFIs within 28 days after T1 according to the route of administration, as well as the duration and maximum level of severity ever experienced over the 28 days following T1 for each S-AEFI and LIS-AEFI, according to the abovementioned grading (none to severe).

The raw proportions of participants whose maximum reported AEFIs was none, mild, moderate, or severe are also shown by route of administration. We also computed the odds ratio (OR) of the maximum severity level of AEFIs experienced by the administration route using multinomial logistic regression models, both univariable and after adjusting for age and HIV status. In these models, the never-reported AEFIs category was chosen as the reference group, and the estimated ORs show the risk of reporting mild, moderate, or severe AEFIs as the maximum level ever experienced vs. none according to the administration route. For a proportion of participants, the diary data were censored at day six, so we also performed a sensitivity analysis after restricting to only the level of AEFI severity ever experienced over the six days following T1.

As a second continuous outcome, we calculated the average number of days in which participants experienced each of the 4 levels of symptoms over the 28 days following T1. We then compared the average duration in days of any systemic or local reactions by route of administration by means of an unpaired t-test. Also, in this analysis, we compared the mean duration of any grade of reaction (from mild to severe, grade 1 to 3) and after restricting to moderate/severe grades (2 or 3). For these analyses with continuous outcomes, instead of using a standard regression model adjusted for covariates (like for the categorical endpoint above), similarly to what is performed for the main immunogenicity response analysis, we aimed to emulate a randomized comparison of the ID vs. SC first dose strategy. Specifically, we calculated counterfactual estimates of what would have been the average duration of any adverse response to the MVA-BN vaccine had everybody in the sample received the ID as first dose vs. everybody having received the SC route of administration instead (ATE).

## 3. Results

### 3.1. Characteristics of the Study Population

Between 8 August and 31 December 2022, 3296 individuals received at least one dose of MVA-BN, of which 654 (19.8%) received the first dose via the SC route and 2642 (80.2%) via the ID route (see Supplementary Figure S1).

Among these, 1008 (30.6%) agreed to be enrolled, either completing the symptom diary and/or accepting to consent blood collection at T1 and T2. Supplementary Table S1 shows the characteristics of these 1008 subjects, of which 269 (26.7%) were vaccinated through the SC route. Overall, 783 subjects (77.7%) completed the diary, 160 (15.9%) completed the diary and were tested for vaccine immunogenicity, and 65 (6.5%) did not complete the diary but did have blood samples available, which were tested, and the results were included in the immunogenicity study (see Supplementary Figure S1). There was weak evidence for a difference in key outcome predictors between populations according to data availability and contribution, suggesting that random selection due to convenience and cost occurred.

For the analysis, we split the population in two groups: those who could be included in the analysis of diary data (*n* = 943, Group 1) and those with available test results from the blood samples stored 1 month after the complete vaccination cycle (*n* = 225, Group 2).

Among the 943 individuals included in Group 1, 225 (23.9%) received the first dose via the SC route, of whom 26 received a single dose because they were vaccine-experienced (11.6% of this group), and 718 (76.1%) via the ID route; of this latter group, 99 (13.8%) received only the first of the two doses scheduled for the previously unvaccinated.

All were male, and the majority (90.9%) self-identified as MSM. Overall, the median age was 44 years (IQR 36–51), 43 (36–48) years in the SC group, and 45 (38–52) years in the ID, respectively (*p* = 0.15). Regarding other characteristics, 167 participants (17.7%) were on PrEP, 227 (24.1%) reported at least one STI diagnosed within the previous year, and 261 subjects (27.7%) were people living with HIV (PLWH), all on highly active antiretroviral therapy and all with undetectable viraemia (≤50 copies/mL). HIV infection was more prevalent in the SC (35%) vs. the ID group (25%, *p* = 0.0004). In those with HIV, CD4 cell count was lower than 200 cells/µL only in 10 participants (3.8%), while it was higher than 500 cells/µL in 211 (80.8%), with no evidence for a difference between the two groups (*p* = 0.232). In contrast, the data showed evidence of a difference between strategies according to the use of PrEP (25.8% vs. 15.2% for SC and ID groups, respectively, *p* < 0.001), while there was no evidence for a difference in all other examined factors (i.e., sexual orientation, history of STI other than HIV, CD4 counts for those with HIV, and history of smallpox vaccination).

**Figure 1:**
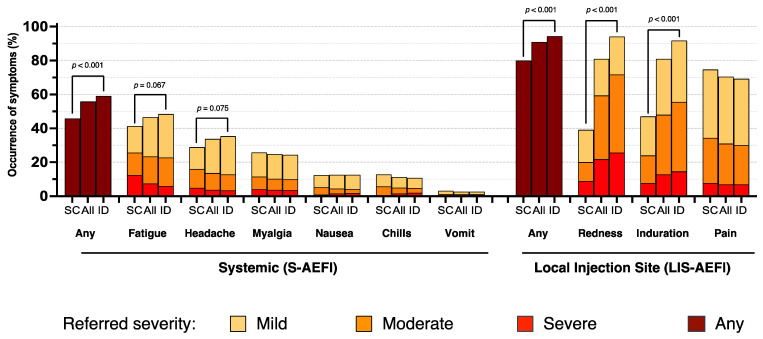
Distribution of reactivity and referred severity of Systemic and Local Injection Site Adverse Effects Following Immunization (S-AEFIs and LIS-AEFIs) with the first dose of MVA-BN vaccine within 28 days from vaccination according to route of administration [All: N = 943; subcutaneous (SC): N = 225; intradermal (ID): N = 718]. *p* < 0.10 are shown; *p* values refer to the difference in the proportion of any grade symptom between the SC and ID groups.

The main characteristics of the study population belonging to Group 1, according to the administration route, are reported in more detail in Table 1. The crude proportions of participants reporting various grades of the evaluated adverse effect are reported in Table 2 and Figure 1.

The grade and duration of adverse events within 28 days from vaccination according to the route of vaccination are instead summarized in Supplementary Figure S2 panel a (S-AEFIs) and Supplementary Figure S2 panel b (LIS-AEFIs).

### 3.2. Systemic Reactions

No serious adverse events were observed over the 28-day follow-up. Overall systemic reactions occurred in 526 (55.9%) participants, with a higher proportion observed in the ID group compared to the SC group (59.1% vs. 45.8%, *p* < 0.001). The most common S-AEFIs were fatigue and headache, occurring in 46.7% and 33.8% of participants, which appeared to be slightly higher in the ID group (*p* = 0.08 and *p* = 0.09, respectively); however, when considering only moderate or severe grades, there was no evidence for a difference between the two groups.

After adjusting for age and HIV status in a multinomial regression model, we found evidence that participants in the ID group had an increased risk of developing mild-grade headaches (2.91; 95% confidence interval, 95% CI: 1.23, 6.89; *p* = 0.045) compared to the SC group (Table 3). Results were similar after restricting the analysis only to the first 6 days of the diary (OR = 2.66, 95% CI: 1.13–6.27, *p* = 0.07), suggesting that most of the difference is likely to occur early after vaccination (Supplementary Table S2).

Systemic reactions were of short duration, 3.7 days on average for any grade and any type of S-AEFI, with no evidence for a difference in symptom duration between the ID and SC groups in the unadjusted analysis and after controlling for HIV and age (Table 4).

### 3.3. Local Reactions

LIS-AEFIs were reported by a total of 852 (90.9%) participants, with a higher proportion in those receiving the ID route (94.4% vs. 80.0%, *p* < 0.001).

Among LIS-AEFIs, redness and induration at the injection site were the most frequently reported adverse effects (80.9% and 81.0%, respectively), with a significantly higher proportion (about twice as high) in the ID group than in the SC group (*p* < 0.001); a similar difference was also observed considering only moderate- or severe-grade adverse events (Table 2).

In contrast, 74.7% of participants in the SC group reported any-grade pain at the injection site vs. 69.2% in the ID group, although there was no evidence for a difference (*p* = 0.14); the same applied when considering moderate or severe grade pain (34.2% vs. 30.1%, *p* = 0.13).

After controlling for HIV and age, we found a higher risk of occurrence for any grade of redness and induration at the local site in the ID than in the SC group (*p* < 0.001). Conversely, a higher degree of pain was reported by participants who received the vaccine through the SC modality (*p* < 0.002). ORs with 95% CI from fitting the multinomial regression models are reported in Table 3. Results were similar after restricting the diary data analysis days 0–6 (Supplementary Table S2).

Local redness and induration (regardless of grade) were the most long-lasting symptoms, especially in the ID group (on average, 18.7 and 16.6 days in ID vs. 5.9 and 5.5 in the SC group, respectively).

After controlling for HIV status and age, we estimated that the duration of redness (regardless of grade) in participants who received the vaccine in ID modality was 12.8 days (95% CI: 9.96, 15.55; *p* < 0.0001) longer than that of subjects receiving the SC strategy. Similar results were observed for induration, which lasted on average 11.1 days (95% CI: 8.45, 13.7; *p* < 0.0001) longer in the ID vs. SC strategy. After restricting the analysis to only moderate or severe grades, we observed a shorter duration of these AEFIs, although still significantly longer in the ID vs. SC group (7.14 vs. 4.24 days for redness, *p* = 0.023 and 5.55 vs. 3.23 days for induration, *p* = 0.004). Overall, local pain (regardless of grade) had a shorter duration, not exceeding 7 days on average, but still with a longer duration in the ID vs. SC group (mean difference 1.8 days (95% CI: 0.2, 3.4; *p* = 0.025) in the unadjusted analysis, which was however largely attenuated after considering only moderate and severe grade and controlling for age and HIV status (*p* = 0.312, Table 4).

### 3.4. Immunogenicity

Finally, Group 2 consisted of 225/1008 (22.3%) vaccine recipients for whom samples were stored and analyzed. In this population, we compared the average change in immunogenicity one month after the completion of the vaccination cycle according to the route of administration (ID vs. SC) both using the raw data and using a counterfactual marginal model.

Within Group 2, the proportion of primed participants was higher in those who received the vaccine through the ID vs. the SC route (47.6% vs. 33.3%, *p* = 0.03, Table 5). There was no evidence for an imbalance in other factors between strategies received under the natural course.

**Figure 2:**
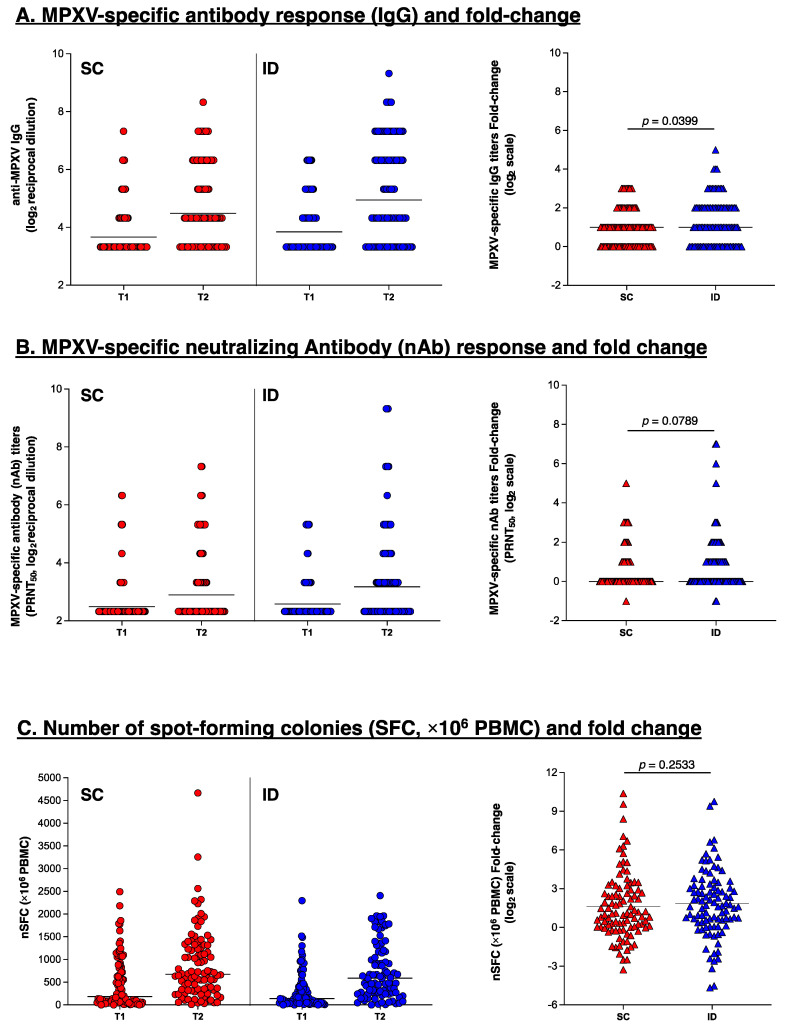
MPXV-specific antibody response (IgG, Panel (**A**)), MPXV-specific neutralizing antibody response (nAb, Panel (**B**)), and frequency of T-cells responding to MVA-BN vaccine expressed as the number of forming colonies (SFC × 10^6^ PBMC) tested by interferon-γ ELISpot assay (Panel (**C**)) in subcutaneous administration (SC, red dots) vs. intradermal administration (ID, blue dots). Panel (**A**): titers of MPXV-specific IgG were measured by immunofluorescence (1:20) starting dilution. Panel (**B**): titers of MPXV-specific nAbs were measured by 50% plaque reduction neutralization test (PRNT_50_, 1:10 starting dilution). Titers in Panels (**A**,**B**) are expressed as geometric mean titers (GMTs) of the reciprocal serum dilution (log_2_ scale); the horizontal lines refer to the GMT. Panel (**C**): Kinetics of T-cell-specific response after stimulation with MVA-BN expressed as number of SFC × 10^6^ PBMC. The horizontal lines refer to the median. On the right part of each panel, fold-change in antibody titers or number of SFCs (on the log_2_ scale) are shown. Mann–Whitney’s test was used for statistical comparison. The horizontal lines refer to the median.

The crude analysis of the raw immunogenicity data showed some evidence for a larger increase in MPXV-specific IgG (GMT, *p* = 0.040) and nAb titers (GMT, *p* = 0.079) in favor of the ID vs. SC administration, while weaker evidence for a difference by administration route was found in the variation in MVA-BN-specific T-cell response measured by ELISpot assay (*p* = 0.253) (see Figure 2).

The results were confirmed by our trial emulation analysis weighted for age and HIV status, showing the following mean differences in the log_2_ scale: (0.26 log_2_, *p* = 0.05 for MPXV-specific IgG) and (0.19 log_2_, *p* = 0.24 for nAb titers) and (0.25 log_2_, *p* = 0.49 for MVA-BN-specific T-cell response measured by ELISpot assay, Table 6). In a sub-analysis performed after stratification according to HIV-status, although the difference in IgG appeared attenuated in participants without HIV (0.15 log_2_ vs. 0.38 log_2_), there was little evidence for effect measure modification from performing the formal interaction test in a standard linear regression model (*p* = 0.60) (see Supplementary Table S3).

Finally, to rule out the potential confounding effect of a boosted immune response in previously vaccinated participants, the analysis was restricted to the primed participants (N = 90) using the variation obtained after receiving the first dose alone as an endpoint, showing results that were similar to those of the main analysis although lacking in statistical power (see Supplementary Table S4).

## 4. Discussion

In our study population, the ID route of administration of the MVA-BN vaccine was safe and well tolerated despite higher reactogenicity than with the SC route. We did not observe serious adverse effects or syncope, as recently reported by the manufacturer [13]. However, among the participants receiving the ID vaccine, we observed a slightly higher prevalence of headache and fatigue than in the SC group. This difference, already mild in the unadjusted analysis, was further attenuated after controlling for the potential confounding effect of age and HIV status. Local redness and induration were confirmed to be more prevalent (94%) and long-lasting (around 18 days) among participants receiving the ID route than those receiving the SC route. On the contrary, pain was more common after SC administration. These findings are quite expected considering the modality of inoculation and are consistent with other reports not only for MVA-BN vaccine [14], but also for other vaccines previously described [6,7]. In particular, Frey et al. reported proportions of adverse effects in people receiving the ID dose of 97% for local and 79% for systemic symptoms, and no severe grade events. Moreover, local symptoms lasted over a month after intradermal administration [14].

Although expected and not severe, redness and induration in the forearm were not well accepted by vaccinated people because they were considered a mark of vaccination and, consequently, of sexual behavior associated with a high risk of contracting mpox.

Stigma could represent a barrier to vaccination, especially in countries where discrimination and racism are deep-rooted and the LGBT community is criminalized [29]. Luckily, MVA-BN can also be inoculated intradermally into the upper back just below the shoulder blade or into the skin of the shoulder above the deltoid muscle [30], where the spot is less visible than in the forearm. Knowledge of these data might help clinicians provide more appropriate counselling, make people aware of the course of side effects, and, therefore, make vaccination more acceptable and sufficiently widespread enough to protect the “core group” who sustains the virus transmission and contributes to its possible spread to the general population [31].

Regarding immunogenicity, our data collected one month after completing the vaccination cycle showed a substantially equivalent immune response between the participants receiving the first dose intradermally and those receiving it subcutaneously. Differently from previously published data, which showed humoral immune response against vaccinia virus (VACV, not MPXV) [17], we measured both titers of specific IgG and nAbs against MPXV, and we found that slightly higher titers of MPXV IgG and nAbs were elicited after the homologous (ID+ID) course of vaccination that after the heterologous (SC+ID) one.

These findings may at least partly explain the results of a report from a study conducted from May 2022 to May 2024 in the USA, showing a higher incidence of breakthrough infections among people fully vaccinated with the heterologous than the homologous ID vaccination cycle. However, of note, in our cohort, we did not observe breakthrough infections [32].

Finally, ours is the first analysis providing data regarding the cellular immune response according to the administration route of MVA-BN. There was no evidence for a difference in the cellular immune response between participants receiving the first dose intradermally or subcutaneously. T-cell response has been known to be crucial for the control and resolution of poxvirus infections [33,34], and T-cell reactivity to VACV and MPXV was detected decades post-vaccination with the first-generation vaccine, suggesting a role of long-lasting cross-reactive T-cell memory responses in vaccine efficacy [35]. A good T-cell response was also demonstrated after third-generation MVA-BN [36]. T-cells elicited from VACV-based vaccines were found to recognize MPXV-derived epitopes, suggesting that it is crucial for the cross-reactivity between different orthopox strains [37] and corelates with mpox clearance [38]. Consequently, T-cells could contribute to the recognition of the different MPXV clades and to broader vaccine efficacy. Interestingly, we found no evidence for a difference in the cellular immune response between participants receiving the first dose intradermally or subcutaneously and therefore our data are consistent, with no benefit in using one strategy over the other to achieve cross-reactive impact.

Recently, the AFRO-Mpox bulletin showed a spreading outbreak involving several African countries, and clade Ib and clade IIb have been sequenced [39]. On balance, although confirmatory studies performed in people infected with clade Ib are needed, in the wake of our reactogenicity and IgG and nAb responses data, we support the use of vaccination by MVA-BN administered by an ID route in low-income countries, as already recommended by the WHO [19]. The strong advantage of the use of intradermal strategy for vaccination lies in the fact that the sparing-dose protocol of ID-based vaccination provides a cost-effective approach to the current global vaccination campaign and could allow for an increase in vaccination coverage, especially in high mpox prevalence areas in low-income countries.

Before drawing firm conclusions, some limitations need to be stated: First, this study’s observational design could not account for unmeasured bias, although the analysis was controlled for the main measured confounders. Furthermore, we were not able to compare the two homologous cycles (SC+SC vs. ID+ID) because the second doses were only administered intradermally due to regulatory recommendations. For this reason, for the diary data, we limited the comparison to the first dose, while for the immunogenicity response, we compared the full cycles, SC+ID vs. ID+ID. Although we found a difference in IgG and nAb response—which was not consistent with the null hypothesis of no difference by strategy and potentially reflects the causal effect of using ID vs. SC as first dose—it is difficult to attach a clinical meaning in terms of risk of infection to the magnitude of the difference found. As mentioned, although we extensively assessed the immune response against MPXV clade IIb, we cannot at this stage provide information on the humoral and cellular response against clade I. Finally, we described the early response after vaccination, and further studies are needed to assess the persistence of immunity according to the administration route.

## 5. Conclusions

In conclusion, our study shows that the ID route of administration of MVA-BN appears to elicit higher titers of MPXV-specific IgG and nAbs than the SC route. At the same time, we found no evidence for a difference in cellular response by strategy. MVA-BN was globally well tolerated despite higher reactogenicity using the ID than the SC route. Based on these results, we believe that ID administration of MVA-BN is feasible, safe, and immunogenic, and our data support the use of this dose-sparing strategy to increase the feasibility of a broad vaccination campaign to control the current multiclade mpox outbreak in Africa.

## Figures and Tables

**Table 1:** Main characteristics of participants who completed the symptom-reporting daily diary covering their experience within 28 days from the first MVA-BN dose administration.

Characteristics		Total N = 943	Route of Administration of the First Vaccination Dose		
			ID N = 718	SC N = 225	*p*-Value *
Sexual orientation, n (%)	Bisexual	65 (6.9)	52 (7.2)	13 (5.8)	0.479
	Transgender	12 (1.3)	9 (1.3)	3 (1.3)	
	MSM	857 (90.9)	648 (90.3)	209 (92.9)	
Age, years, median (IQR)		44 (36, 51)	45 (38, 52)	43 (36, 48)	0.153
PrEP use, n (%)	No	759 (80.5)	604 (84.1)	155 (68.9)	<0.001
	Yes	167 (17.7)	109 (15.2)	58 (25.8)	
	Not reported	17 (1.8)	5 (0.7)	12 (5.3)	
≥1 STI over previous year, n (%)	Yes	209 (22.4)	152 (21.2)	57 (25.3)	0.190
	Syphilis	108 (57.4)	72 (55.0)	36 (63.2)	0.222
	Gonorrhea	73 (39.9)	44 (34.9)	29 (50.9)	0.329
	Chlamydia	43 (24.6)	22 (18.6)	21 (36.8)	0.324
	HPV	28 (16.4)	14 (12.3)	14 (24.6)	0.298
PLWH (all on ART)	Yes	261 (27.7)	182 (25.3)	79 (35.1)	0.004
CD4 cell count ^&^, cells/mm^3^, n (%)	0–200	10 (3.8)	5 (2.7)	5 (6.3)	0.232
	201–500	39 (14.9)	28 (15.4)	11 (13.9)	
	501+	211 (80.8)	149 (81.9)	62 (78.5)	
	Unknown	1 (0.4)	0 (0.0)	1 (1.3)	
Previous smallpox vaccination, n (%)	Yes	125 (13.3)	99 (13.8)	26 (11.6)	0.338

^&^ In PLWH; * chi-square or Mann–Whitney test as appropriate. ID: intradermal; SC: subcutaneous; MSM: men who have sex with men; IQR: interquartile range; PrEP: pre-exposure prophylaxis for HIV infection; STI: sexually transmitted infection; HPV: human papillomavirus; PLWH: people living with HIV; ART: antiretroviral therapy.

**Table 2:** Table of prevalence of Systemic and Local Adverse Effects Following Immunization (S-AEFIs and LIS-AEFIs) with MVA-BN vaccine.

			Prevalence of Symptoms (ID vs. SC)		
Adverse Effect	Grade	Tot n (%)	ID n (%)	SC n (%)	*p*-Value *
Any S-AEFI	No	415 (44.1)	293 (40.9)	122 (54.2)	
	Yes, any grade	526 (55.9)	423 (59.1)	103 (45.8)	<0.001
Fatigue	No	502 (53.3)	370 (51.7)	132 (58.7)	
	Yes, any grade	439 (46.7)	346 (48.3)	93 (41.3)	0.079
	Yes, moderate or severe	221 (23.5)	163 (22.8)	58 (25.8)	0.938
Headache	No	623 (66.2)	463 (64.7)	160 (71.1)	
	Yes, any grade	318 (33.8)	253 (35.3)	65 (28.9)	0.089
	Yes, moderate or severe	128 (13.6)	92 (12.8)	36 (16)	0.644
Myalgia	No	708 (75.2)	541 (75.6)	167 (74.2)	
	Yes, any grade	233 (24.8)	175 (24.4)	58 (25.8)	0.752
	Yes, moderate or severe	97 (10.3)	71 (9.9)	26 (11.6)	0.569
Nausea	No	823 (87.5)	626 (87.4)	197 (87.6)	
	Yes, any grade	118 (12.5)	90 (12.6)	28 (12.4)	0.948
	Yes, moderate or severe	42 (4.5)	30 (4.2)	12 (5.3)	0.617
Chills	No	835 (88.7)	639 (89.2)	196 (87.1)	
	Yes, any grade	106 (11.3)	77 (10.8)	29 (12.9)	0.446
	Yes, moderate or severe	47 (5)	34 (4.7)	13 (5.8)	0.631
Vomit	No	915 (97.2)	697 (97.3)	218 (96.9)	
	Yes, any grade	26 (2.8)	19 (2.7)	7 (3.1)	0.895
	Yes, moderate or severe	11 (1.2)	8 (1.1)	3 (1.3)	0.999
Any LIS-AEFI	No	85 (9.1)	40 (5.6)	45 (20.0)	
	Yes, any grade	852 (90.9)	672 (94.4)	180 (80.0)	<0.001
Redness	No	179 (19.1)	42 (5.9)	137 (60.9)	
	Yes, any grade	758 (80.9)	670 (94.1)	88 (39.1)	<0.001
	Yes, moderate or severe	556 (59.3)	511 (71.8)	45 (20.0)	<0.001
Induration	No	178 (19.0)	59 (8.3)	119 (52.9)	
	Yes, any grade	759 (81.0)	653 (91.7)	106 (47.1)	<0.001
	Yes, moderate or severe	450 (48.0)	396 (55.6)	54 (24.0)	<0.001
Pain	No	276 (29.5)	219 (30.8)	57 (25.3)	
	Yes, any grade	661 (70.5)	493 (69.2)	168 (74.7)	0.141
	Yes, moderate or severe	291 (31.1)	214 (30.1)	77 (34.2)	0.127

* Chi-square or Fisher’s exact test as appropriate, comparing No vs. Yes, any grade, or No vs. Yes, moderate or severe.

**Table 3:** Prevalence and risk of developing different grades of Systemic and Local Adverse Effects Following Immunization (S-AEFIs and LIS-AEFIs) with MVA-BN vaccine from fitting a multinomial logistic regression according to administration route: intradermal (ID) vs. subcutaneous (SC).

		Prevalence of S-AEFIs and OR (ID vs. SC)					
Systemic Symptoms	Max Severity	ID n (%)	SC n (%)	Unadjusted OR (95% CI) ID vs. SC	*p*-Value	Adjusted * OR (95% CI) ID vs. SC	*p*-Value
Fatigue	None	370 (51.7)	132 (58.7)	1	<0.001	1	0.673
	Mild	183 (25.6)	35 (15.6)	1.87 (1.23, 2.82)		0.95 (0.40, 2.24)	
	Moderate	120 (16.8)	30 (13.3)	1.43 (0.91, 2.23)		1.67 (0.66, 4.26)	
	Severe	43 (6.0)	28 (12.4)	0.55 (0.33, 0.92)		1.54 (0.49, 4.81)	
Headache	None	463 (64.7)	160 (71.1)	1	0.011	1	0.045
	Mild	161 (22.5)	29 (12.9)	1.92 (1.24, 2.96)		2.91 (1.23, 6.89)	
	Moderate	68 (9.5)	25 (11.1)	0.94 (0.57, 1.54)		1.37 (0.44, 4.27)	
	Severe	24 (3.4)	11 (4.9)	0.75 (0.36, 1.57)		7.17 (0.74, 69.81)	
Myalgia	None	541 (75.6)	167 (74.2)	1	0.915	1	0.675
	Mild	104 (14.5)	32 (14.2)	1.00 (0.65, 1.55)		1.04 (0.39, 2.78)	
	Moderate	45 (6.3)	17 (7.6)	0.82 (0.46, 1.47)		1.27 (0.37, 4.32)	
	Severe	26 (3.6)	9 (4.0)	0.89 (0.41, 1.94)		2.47 (0.57, 10.68)	
Nausea	None	626 (87.4)	197 (87.6)	1	0.294	1	0.723
	Mild	60 (8.4)	16 (7.1)	1.18 (0.66, 2.10)		1.79 (0.50, 6.46)	
	Moderate	17 (2.4)	10 (4.4)	0.53 (0.24, 1.19)		0.55 (0.10, 3.13)	
	Severe	13 (1.8)	2 (0.9)	Nd		Nd	
Chills	None	639 (89.2)	196 (87.1)	1	0.089	1	0.698
	Mild	43 (6.0)	16 (7.1)	0.82 (0.45, 1.50)		1.13 (0.37, 3.50)	
	Moderate	20 (2.8)	12 (5.3)	0.51 (0.25, 1.06)		0.27 (0.03, 2.48)	
	Severe	14 (2.0)	1 (0.4)	Nd		Nd	
Vomit	None	697 (97.3)	218 (96.9)	1	0.195	1	0.748
	Mild	11 (1.5)	4 (1.8)	0.86 (0.27, 2.73)		2.68 (0.21, 33.94)	
	Moderate	3 (0.4)	3 (1.3)	Nd		Nd	
	Severe	5 (0.7)	0 (0.0)	Nd		Nd	
		**Prevalence of LIS-AEFIs and OR (ID vs. SC)**					
**Local Symptoms**	**Max Severity**	**ID** **n (%)**	**SC** **n (%)**	**Unadjusted OR (95% CI)** **ID vs. SC**	***p*-Value**	Adjusted * OR (95% CI) **ID vs. SC**	***p*-Value**
Redness	None	42 (5.9)	137 (60.9)	1	0.089	1	<0.001
	Mild	159 (22.3)	43 (19.1)	12.06 (7.44, 19.54)		26.87 (6.87, 105.1)	
	Moderate	328 (46.1)	25 (11.1)	42.80 (25.10, 72.98)		65.91 (16.39, 265.1)	
	Severe	183 (25.7)	20 (8.9)	29.85 (16.77, 53.13)		48.44 (11.71, 200.4)	
Induration	None	59 (8.3)	119 (52.9)	1	<0.001	1	<0.001
	Mild	257 (36.1)	52 (23.1)	9.97 (6.47, 15.35)		12.08 (4.44, 32.89)	
	Moderate	292 (41.0)	37 (16.4)	15.92 (10.02, 25.29)		14.92 (5.03, 44.22)	
	Severe	104 (14.6)	17 (7.6)	12.34 (6.77, 22.49)		16.28 (4.73, 55.98)	
Pain	None	219 (30.8)	57 (25.3)	1	0.425	1	0.002
	Mild	279 (39.2)	91 (40.4)	0.80 (0.55, 1.16)		0.36 (0.16, 0.81)	
	Moderate	165 (23.2)	60 (26.7)	0.72 (0.47, 1.08)		0.14 (0.05, 0.39)	
	Severe	49 (6.9)	17 (7.6)	0.75 (0.40, 1.40)		0.57 (0.15, 2.18)	

* Adjusted for age and HIV status. 95% CI: 95% confidence interval. Nd: not determined.

**Table 4:** Contrasts of the duration of Systemic and Local Adverse Effects Following Immunization (S-AEFIs and LIS-AEFIs) with MVA-BN vaccine (days 1–28 after 1st dose) and ATE ^&^ from fitting a linear regression model.

	Potential Average Duration of Systemic Symptoms (Days) over 1–28 Days After the First Vaccine Dose			
	Mean in ID (95% CI)	Mean in SC (95% CI)	ATE * (95% CI) ID vs. SC	*p*-Value
**S-AEFIs**				
Fatigue—any	3.70 (2.63, 4.77)	3.46 (2.21, 4.72)	0.24 (−1.36, 1.84)	0.7720
Fatigue—moderate or severe	2.95 (1.26, 4.63)	3.03 (1.07, 4.98)	−0.08 (−2.52, 2.36)	0.9488
Headache—any	2.20 (1.53, 2.87)	2.51 (1.81, 3.20)	−0.30 (−1.24, 0.63)	0.5269
Headache—moderate or severe	2.53 (0.99, 4.07)	2.20 (1.23, 3.16)	0.34 (−1.37, 2.04)	0.7005
Myalgia—any	3.65 (2.11, 5.19)	3.12 (2.14, 4.10)	0.52 (−1.21, 2.25)	0.5527
Myalgia—moderate or severe	3.20 (0.51, 5.90)	2.17 (0.71, 3.63)	1.03 (−1.64, 3.70)	0.4479
Nausea—any	3.31 (1.61, 5.00)	1.74 (1.17, 2.30)	1.57 (−0.09, 3.22)	0.0632
Chills—any	2.10 (1.32, 2.88)	1.92 (1.07, 2.77)	0.18 (−0.85, 1.21)	0.7289
**LIS-AEFIs**				
Redness—any	18.66 (16.76, 20.55)	5.90 (3.72, 8.09)	12.76 (9.96, 15.55)	<0.0001
Redness—moderate or severe	7.14 (5.61, 8.66)	4.24 (2.06, 6.42)	2.90 (0.41, 5.39)	0.0226
Induration—any	16.62 (14.51, 18.73)	5.54 (3.66, 7.41)	11.09 (8.45, 13.72)	<0.0001
Induration—moderate or severe	5.55 (4.08, 7.02)	3.23 (2.66, 3.81)	2.32 (0.75, 3.89)	0.0037
Pain—any	6.18 (4.66, 7.70)	4.37 (3.86, 4.88)	1.81 (0.22, 3.39)	0.0254
Pain—moderate or severe	4.77 (1.69, 7.85)	3.13 (2.54, 3.72)	1.64 (−1.54, 4.82)	0.3124

^&^ average treatment effect; * weighted for age and HIV status.

**Table 5:** Main characteristics of participants for whom samples were stored and analyzed to assess immunogenicity, by route of administration.

**Characteristics**		**Total** **N = 225**	**Route of Administration of the First Vaccination Dose**		
			**ID** **N = 105**	**SC** **N = 120**	*p*-Value *
Sexual orientation, n (%)	Bisexual	17 (7.6%)	8 (7.6%)	9 (7.5%)	0.682
	Transgender	4 (1.8%)	1 (1.0%)	3 (2.5%)	
	MSM	204 (90.7%)	96 (91.4%)	108 (90.0%)	
Age, years, Median (IQR)		45 (36, 52)	46 (38, 52)	44 (35, 51)	0.164
PLWH (all on ART)	Yes	109 (48.4)	49 (46.7)	60 (50.0)	0.618
CD4 cell count ^&^, cells/mm^3^, n (%)	0–200	5 (4.6%)	3 (6.1%)	2 (3.3%)	0.759
	201–500	17 (15.6%)	7 (14.3%)	10 (16.7%)	
	501+	87 (79.8%)	39 (79.6%)	48 (80.0%)	
Previous smallpox vaccination, n (%)	Yes	90 (40.0)	50 (47.6)	40 (33.3)	0.029

^&^ in PLWH; * chi-square or Mann–Whitney test as appropriate. ID: intradermal; SC: subcutaneous; MSM: men who have sex with men; IQR: interquartile range; PLWH: people living with HIV; ART: antiretroviral therapy.

**Table 6:** Potential average change one month after the completion of vaccination cycle according to the route of administration of the first dose and ATE ^&^ from fitting a marginal model (log_2_ scale).

	Mean (log_2_) in ID (95% CI)	Mean (log_2_) in SC (95% CI)	ATE * (95% CI)	*p*-Value
anti-MPXV IgG	1.22 (1.00, 1.43)	0.96 (0.80, 1.13)	0.26 (−0.00, 0.51)	0.053
anti-MPXV nAbs	0.76 (0.50, 1.02)	0.57 (0.39, 0.75)	0.19 (−0.13, 0.51)	0.244
SFC (ELISpot)	1.86 (1.33, 2.39)	1.61 (1.11, 2.11)	0.25 (−0.45, 0.94)	0.491

^&^ average treatment effect; nAbs: anti-MPXV neutralizing antibodies; SFC: spot-forming colonies (×10^6^ PBMC); * weighted for age, HIV-status, and previous smallpox vaccination.

## Data Availability

Data are available upon reasonable request from the corresponding author.

## Supplementary Materials

The following supporting information can be downloaded at: https://www.mdpi.com/article/10.3390/vaccines13010032/s1, Supplementary Figure S1. Flow-chart showing overall vaccinated subjects and study participants, according to route of administration of the first MVA-BN dose (subcutaneous, SC or intradermal, ID route) and availability of data (from symptom-reporting daily diary and/or data from blood samples). Supplementary Table S1. Main characteristics of participants according to availability of data from symptom-reporting daily diary and/or data from blood samples. Supplementary Figure S2. Heatmap summarizing grade and duration of Systemic (Panel a) and Local Injection Site (Panel b) Adverse Effects Following Immunization (S-AEFI and LIS-AEFI) with MVA-BN vaccine within 28-days from vaccination according to route of vaccination [subcutaneous (SC): N = 225; intradermal (ID): N = 718]. Every row represents results for a participant between Days 1 and 28, which are presented as columns. Cells are color-coded by severity. Supplementary Table S2: Prevalence and risk of developing different grades of Systemic and Local Adverse Effects Following Immunization (S-AEFIs and L-AEFIs) with MVA-BN vaccine up to six days from fitting a multinomial logistic regression according to administration route—intradermal (ID) vs. subcutaneous (SC). Supplementary Table S3. Potential average change one month after the completion of vaccination cycle according to the route of administration of the first dose and average treatment effect (ATE) from fitting a marginal regression model (log2 scale), according to HIV status (PLWH N = 109/225). Supplementary Table S4. Potential average change one month after the completion of vaccination cycle according to the route of administration of the first dose and average treatment effect (ATE) from fitting a marginal model (log2 scale): analysis restricted the response to first dose alone in participants primed with smallpox vaccine (N = 90, of which 40 through SC route and 50 through ID route).
