# Supplementary material for: Reactogenicity and Immunogenicity Against MPXV of the Intradermal Administration of Modified Vaccinia Ankara Compared to the Standard Subcutaneous Route
Source: Vaccines (Basel). 2024 Dec 31;13(1):32. doi: 10.3390/vaccines13010032 (PMC11769009; doi:10.3390/vaccines13010032)
Supplement: Supplementary file 1 [file vaccines-13-00032-s001.zip › Suppl_Figure_S1.pptx]

## Slide 1
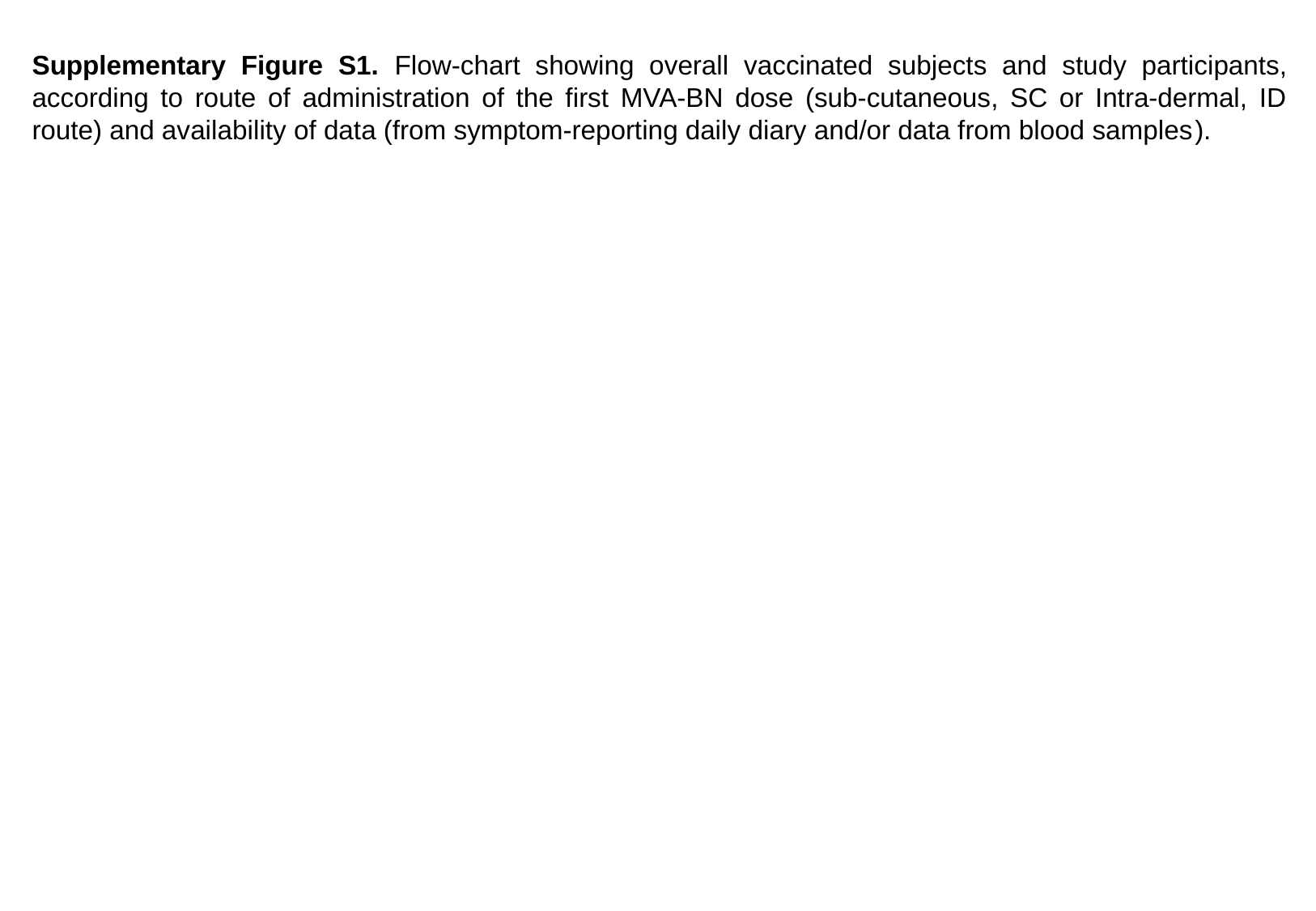

Supplementary Figure S1. Flow-chart showing overall vaccinated subjects and study participants, according to route of administration of the first MVA-BN dose (sub-cutaneous, SC or Intra-dermal, ID route) and availability of data (from symptom-reporting daily diary and/or data from blood samples).

## Slide 2
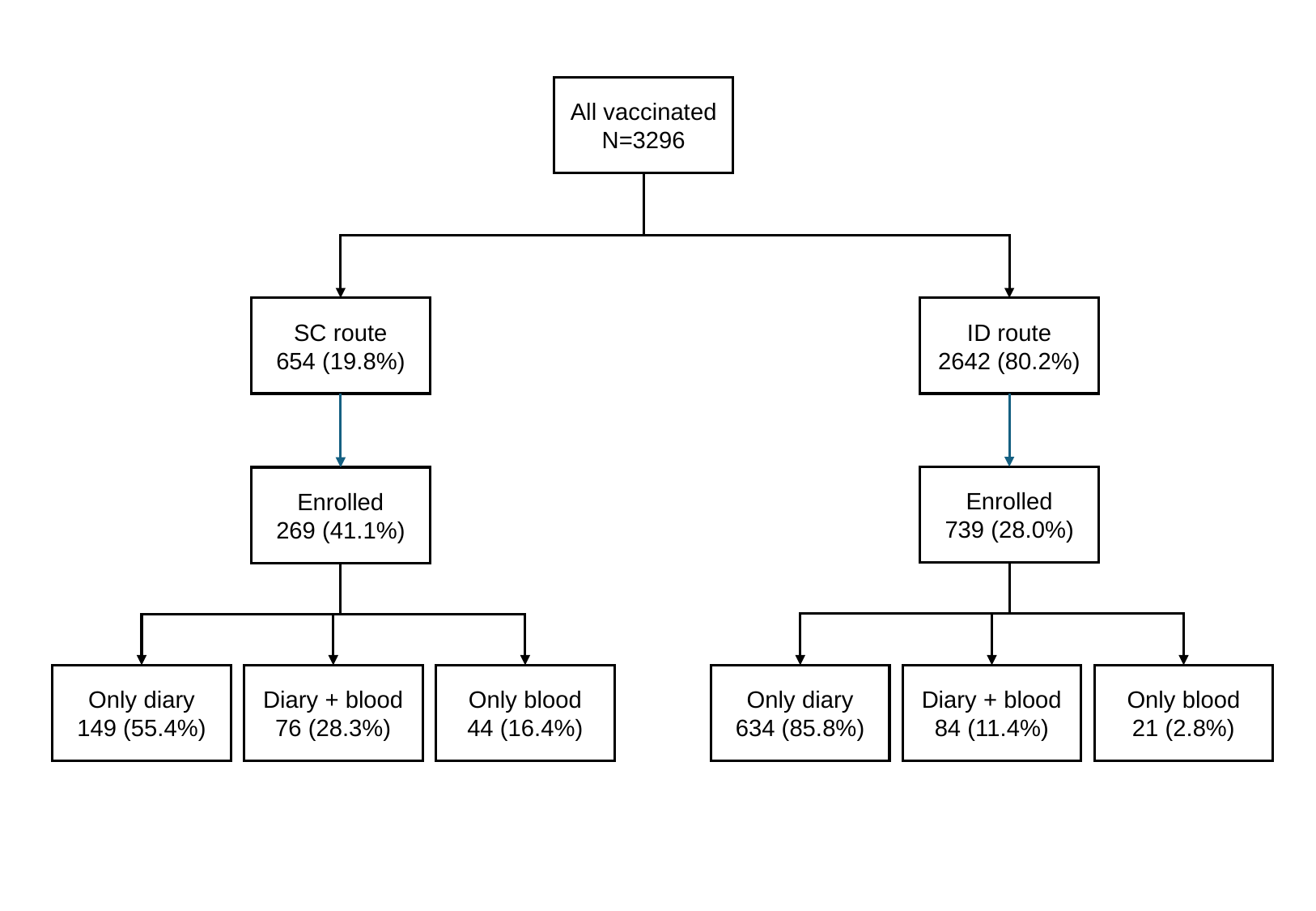

All vaccinated
N=3296
SC route
654 (19.8%)
ID route
2642 (80.2%)
Enrolled
739 (28.0%)
Enrolled
269 (41.1%)
Only diary
149 (55.4%)
Diary + blood
76 (28.3%)
Only blood
44 (16.4%)
Only diary
634 (85.8%)
Diary + blood
84 (11.4%)
Only blood
21 (2.8%)
