# Supplementary material for: Reactogenicity and Immunogenicity Against MPXV of the Intradermal Administration of Modified Vaccinia Ankara Compared to the Standard Subcutaneous Route
Source: Vaccines (Basel). 2024 Dec 31;13(1):32. doi: 10.3390/vaccines13010032 (PMC11769009; doi:10.3390/vaccines13010032)
Supplement: Supplementary file 1 [file vaccines-13-00032-s001.zip › Suppl_Figure_S2.pptx]

## Slide 1
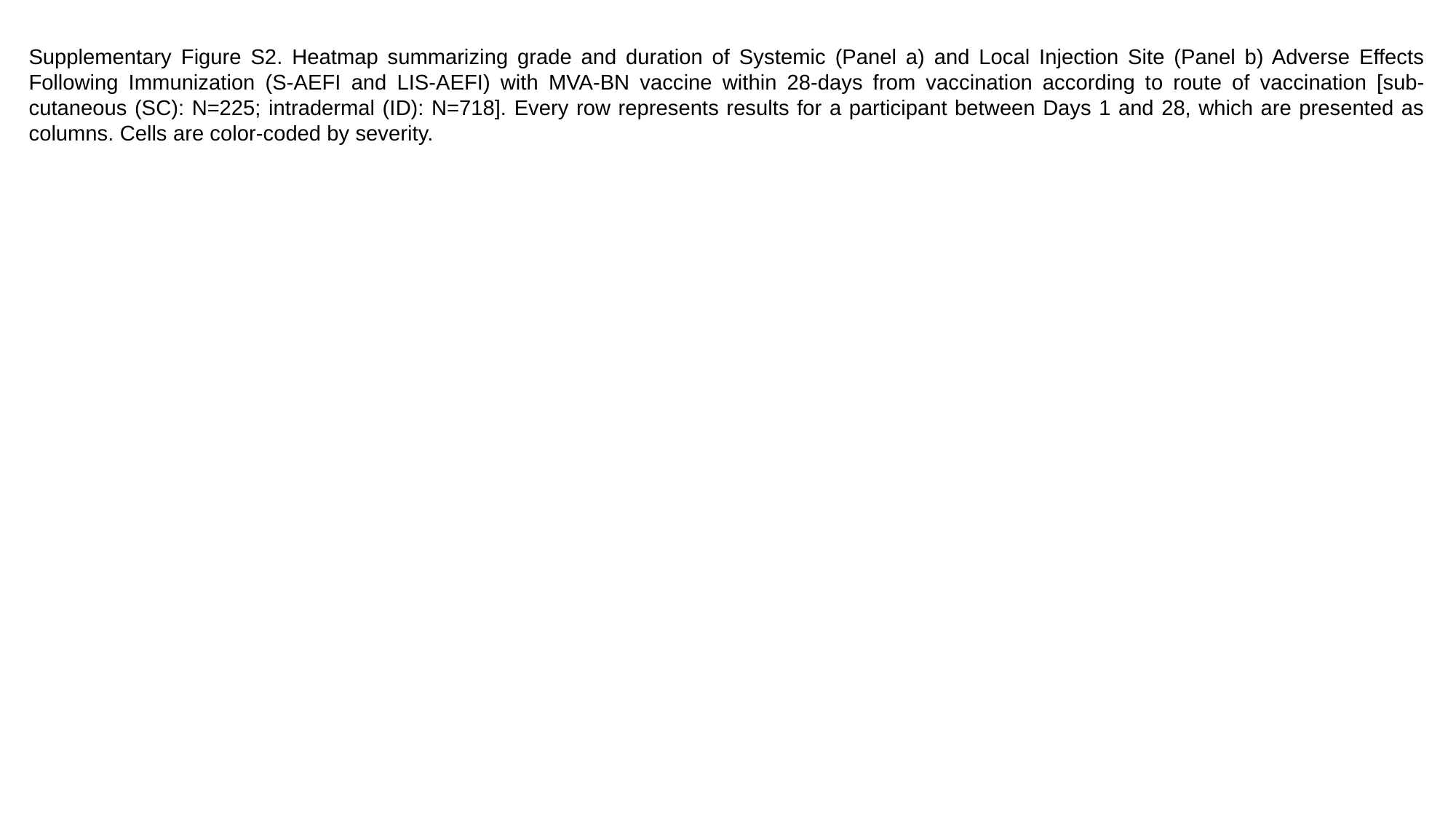

Supplementary Figure S2. Heatmap summarizing grade and duration of Systemic (Panel a) and Local Injection Site (Panel b) Adverse Effects Following Immunization (S-AEFI and LIS-AEFI) with MVA-BN vaccine within 28-days from vaccination according to route of vaccination [sub-cutaneous (SC): N=225; intradermal (ID): N=718]. Every row represents results for a participant between Days 1 and 28, which are presented as columns. Cells are color-coded by severity.

## Slide 2
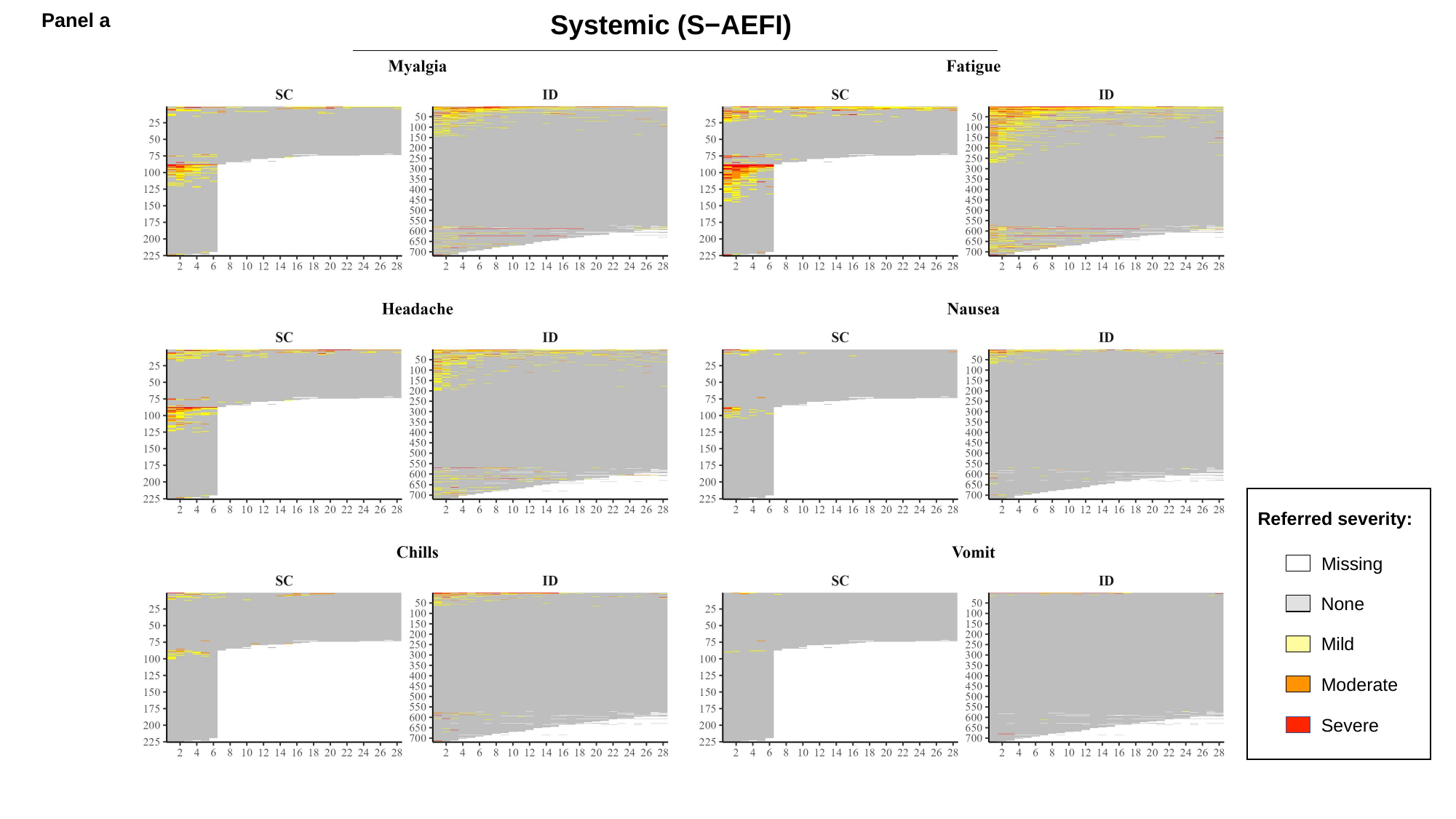

Panel a
Systemic (S−AEFI)
Referred severity:
Missing
None
Mild
Moderate
Severe

## Slide 3
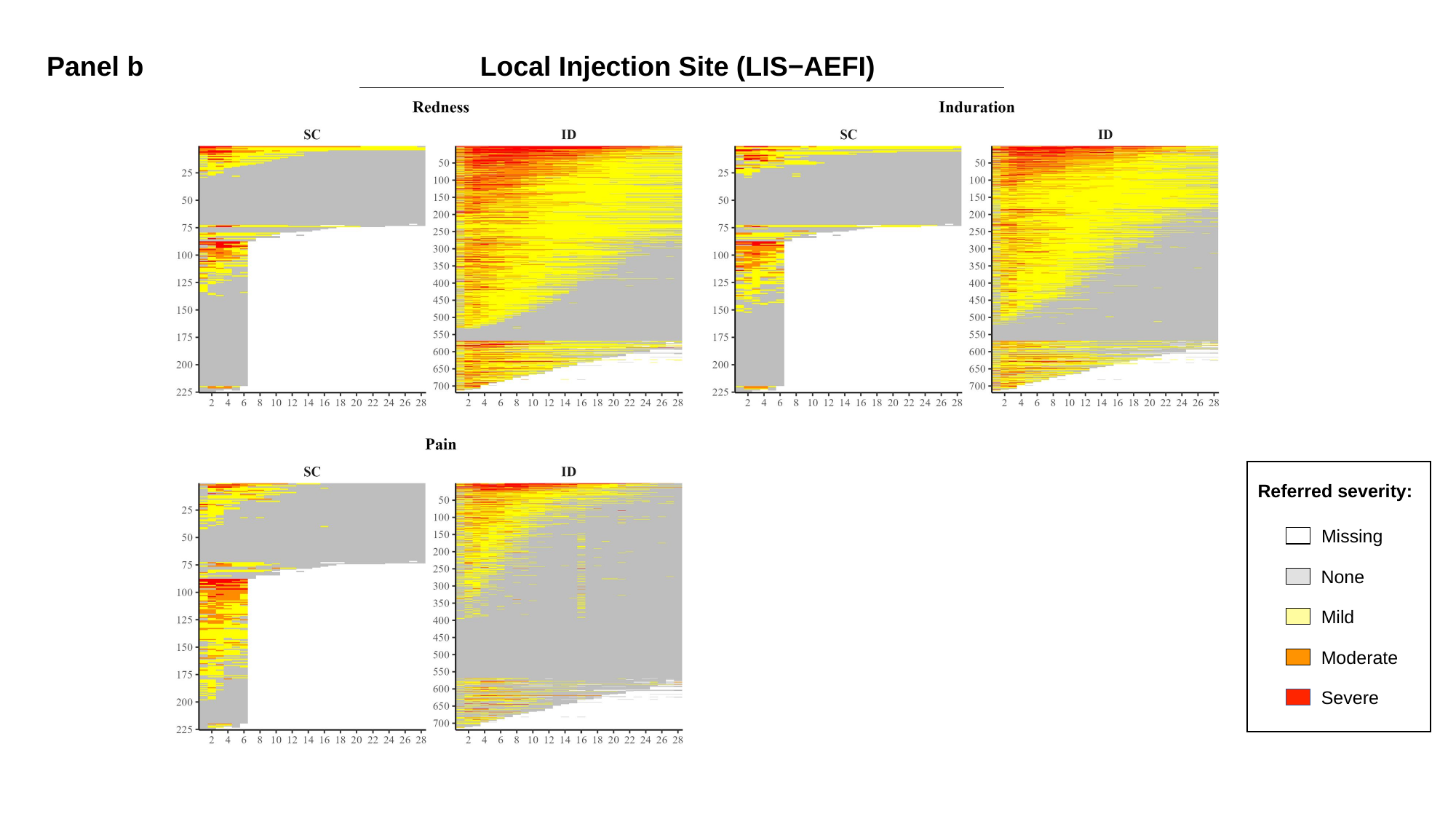

Panel b
Local Injection Site (LIS−AEFI)
Referred severity:
Missing
None
Mild
Moderate
Severe
