# Supplementary material for: Reactogenicity and Immunogenicity Against MPXV of the Intradermal Administration of Modified Vaccinia Ankara Compared to the Standard Subcutaneous Route
Source: Vaccines (Basel). 2024 Dec 31;13(1):32. doi: 10.3390/vaccines13010032 (PMC11769009; doi:10.3390/vaccines13010032)
Supplement: Supplementary file 1 [file vaccines-13-00032-s001.zip › Suppl_Table_S1.pdf]

Supplementary Table S1. Main characteristics of participants according to availability of data from symptom-reporting daily diary and/or data from blood samples.

Characteristics		Total N= 1008	Availability of data			p-value*
			Diary but not blood N= 783	Both blood and diary N=160	Blood but not diary N= 65	
Sexual orientation, n (%)	Bisexual	69 (6.8)	52 (6.6)	14 (8.8)	3 (4.6)	0.481
	Transgender	12 (1.2)	8 (1.0)	4 (2.5)	0 (0.0)	
	MSM	927 (92.0)	723 (92.3)	142 (88.8)	62 (95.4)	
Age, years, Median (IQR)		44 (36, 52)	42 (37, 42)	45 (36, 52)	45 (36, 53)	0.376
PREP use, n (%)	No	803 (79.7)	639 (81.6)	120 (75.0)	44 (67.7)	<0.001
	Yes	183 (18.2)	138 (17.6)	29 (18.1)	16 (24.6)	
	Not reported	22 (2.2)	6 (0.8)	11 (6.9)	5 (7.7)	
≥ 1 STI over previous year, n (%)	Yes	229 (22.7)	181 (23.1)	28 (17.5)	20 (30.8)	0.084
	Siphilis	109 (10.8)	85 (10.9)	14 (8.8)	10 (15.4)	0.347
	Gonorrhoea	72 (7.1)	58 (7.4)	6 (3.8)	8 (12.3)	0.065
	Chlamydia	37 (3.7)	25 (3.2)	9 (5.6)	3 (4.6)	0.302
	HPV	22 (2.2)	16 (2.0)	3 (1.9)	3 (4.6)	0.379
PLWH (all on ART)	Yes	289 (28.7)	180 (23.0)	81 (50.6)	28 (43.1)	<0.001
CD4 cell count[&], cells/mm^3, n (%)	0-200	10 (3.5)	5 (2.8)	5 (6.2)	0 (0.0)	0.453
	201-500	46 (15.9)	29 (16.1)	10 (12.3)	7 (25.0)	
	501+	232 (80.3)	145 (80.6)	66 (81.5)	21 (75.0)	
	Not measured	1 (0.3)	1 (0.6)	0 (0.0)	0 (0.0)	
Previous smallpox vaccination, n (%)	Yes	157 (15.6)	67 (8.6)	58 (36.3)	32 (49.2)	<0.001
Route of administration, n (%)	Sub-cutaneous	269 (26.7)	149 (19.0)	76 (47.5)	44 (67.7)	<0.001
	Intra-dermic	739 (73.3)	634 (81.0)	84 (52.5)	21 (32.3)	

[&] In PLWH; * chi-square or Kruskall-Wallis test as appropriate
MSM: men who have sex with men; IQR: interquartile range; PrEP: pre-exposure prophylaxis for HIV infection; STI: sexually transmitted infection; HPV: human papillomavirus; PLWH: people living with HIV; ART: antiretroviral therapy.
