# Supplementary material for: Reactogenicity and Immunogenicity Against MPXV of the Intradermal Administration of Modified Vaccinia Ankara Compared to the Standard Subcutaneous Route
Source: Vaccines (Basel). 2024 Dec 31;13(1):32. doi: 10.3390/vaccines13010032 (PMC11769009; doi:10.3390/vaccines13010032)
Supplement: Supplementary file 1 [file vaccines-13-00032-s001.zip › Suppl_Table_S2.pdf]

Supplementary Table S2. Prevalence and risk of developing different grades of Systemic and Local Adverse Effects Following Immunization (S-AEFIs and L-AEFIs) with MVA-BN vaccine up to six days from fitting a multinomial logistic regression according to administration route - intradermal (ID) vs subcutaneous (SC).

		Prevalence of S-AEFIs and OR (ID vs. SC) up to 6 days from vaccination					
**Systemic symptoms**	**max severity**	**ID n (%)**	**SC n (%)**	**Unadjusted OR (95% CI) ID vs. SC**	**p-value**	**Adjusted* OR (95% CI) ID vs. SC**	**p-value**
Fatigue	None	401 (56.0)	137 (60.9)	1	0.001	1	0.856
	Mild	174 (24.3)	35 (15.6)	1.70 (1.13, 2.56)		0.85 (0.37, 1.98)	
	Moderate	106 (14.8)	29 (12.9)	1.25 (0.79, 1.97)		1.41 (0.54, 3.69)	
	Severe	35 (4.9)	24 (10.7)	0.50 (0.29, 0.87)		1.20 (0.38, 3.81)	
Headache	None	500 (69.8)	166 (73.8)	1	0.022	1	0.065
	Mild	152 (21.2)	29 (12.9)	1.74 (1.13, 2.69)		2.66 (1.13, 6.27)	
	Moderate	47 (6.6)	22 (9.8)	0.71 (0.42, 1.21)		0.95 (0.25, 3.67)	
	Severe	17 (2.4)	8 (3.6)	0.71 (0.30, 1.66)		6.60 (0.68, 63.98)	
Myalgia	None	567 (79.2)	172 (76.4)	1	0.811	1	0.970
	Mild	92 (12.8)	32 (14.2)	0.87 (0.56, 1.35)		1.03 (0.39, 2.72)	
	Moderate	38 (5.3)	13 (5.8)	0.89 (0.46, 1.70)		1.11 (0.26, 4.73)	
	Severe	19 (2.7)	8 (3.6)	0.72 (0.31, 1.67)		1.47 (0.31, 6.94)	
Nausea	None	646 (90.2)	199 (88.4)	1	0.176	1	0.895
	Mild	49 (6.8)	15 (6.7)	1.01 (0.55, 1.83)		1.20 (0.31, 4.71)	
	Moderate	11 (1.5)	9 (4.0)	0.38 (0.15, 0.92)		0.53 (0.09, 3.02)	
	Severe	10 (1.4)	2 (0.9)	Nd		Nd	
Chills	None	656 (91.6)	202 (89.8)	1	0.260	1	0.803
	Mild	35 (4.9)	15 (6.7)	0.72 (0.38, 1.34)		0.89 (0.25, 3.09)	
	Moderate	13 (1.8)	7 (3.1)	0.57 (0.23, 1.45)		0.31 (0.03, 3.17)	
	Severe	12 (1.7)	1 (0.4)	Nd		Nd	
Vomit	None	710 (99.2)	219 (97.3)	1	0.559	1	0.993
	Mild	3 (0.4)	3 (1.3)	0.31 (0.06, 1.54)		Nd	
	Moderate	0 (0.0)	3 (1.3)	Nd		Nd	
	Severe	3 (0.4)	0 (0.0)	Nd		Nd	

		Prevalence of L-AEFIs and OR (ID vs. SC)					
**Local symptoms**	**max severity**	**ID n (%)**	**SC n (%)**	**Unadjusted OR ID vs. SC**	**p-value**	**Adjusted* OR ID vs. SC**	**p-value**
Redness	None	46 (6.5)	137 (60.9)	1	<.001	1	<0.001
	Mild	168 (23.6)	43 (19.1)	11.63 (7.25, 18.67)		20.84 (6.05, 71.73)	
	Moderate	325 (45.6)	25 (11.1)	38.72 (22.87, 65.54)		47.01 (13.15, 168.0)	
	Severe	173 (24.3)	20 (8.9)	25.76 (14.56, 45.59)		36.37 (9.90, 133.7)	
Induration	None	67 (9.4)	121 (53.8)	1	<.001	1	<0.001
	Mild	264 (37.1)	51 (22.7)	9.35 (6.12, 14.27)		10.84 (4.11, 28.57)	
	Moderate	281 (39.5)	36 (16.0)	14.10 (8.92, 22.28)		12.95 (4.55, 36.83)	
	Severe	100 (14.0)	17 (7.6)	10.62 (5.86, 19.25)		14.20 (4.28, 47.14)	
Pain	None	257 (36.1)	60 (26.7)	1	0.057	1	<0.001
	Mild	266 (37.4)	90 (40.0)	0.69 (0.48, 1.00)		0.21 (0.10, 0.48)	
	Moderate	147 (20.6)	58 (25.8)	0.59 (0.39, 0.90)		0.09 (0.03, 0.26)	
	Severe	42 (5.9)	17 (7.6)	0.58 (0.31, 1.08)		0.37 (0.09, 1.45)	

* Adjusted for age and HIV status; 95 CI: 95% confidence interval; Nd: not determined
