# Supplementary material for: Reactogenicity and Immunogenicity Against MPXV of the Intradermal Administration of Modified Vaccinia Ankara Compared to the Standard Subcutaneous Route
Source: Vaccines (Basel). 2024 Dec 31;13(1):32. doi: 10.3390/vaccines13010032 (PMC11769009; doi:10.3390/vaccines13010032)
Supplement: Supplementary file 1 [file vaccines-13-00032-s001.zip › Suppl_Table_S3.pdf]

Supplementary Table S3. Potential average change one month after the completion of vaccination cycle according to the route of administration of the first dose and average treatment effect (ATE) from fitting a marginal model (log_2 scale), according to HIV-status (PLWH N=109/225).

		Mean (log_2) in ID (95% CI)	Mean (log_2) in SC (95% CI)	ATE* (95% CI)	Interaction p-value$
anti-MPXV IgG	In PLWH	1.31 (1.02, 1.59)	0.92 (0.69, 1.16)	0.38 (0.03, 0.73)	0.60
	In participants without HIV	1.14 (0.81, 1.47)	1.00 (0.77, 1.22)	0.15 (–0.24, 0.53)	
anti-MPXV nAbs	In PLWH	0.72 (0.33, 1.10)	0.67 (0.44, 0.90)	0.05 (–0.39, 0.48)	0.46
	In participants without HIV	0.81 (0.40, 1.22)	0.46 (0.19, 0.73)	0.35 (–0.14, 0.84)	
SFC (ELISpot)	In PLWH	2.10 (1.30, 2.90)	1.89 (1.09, 2.68)	0.21 (–0.91, 1.34)	0.82
	In participants without HIV	1.63 (1.01, 2.26)	1.37 (0.75, 2.00)	0.26 (–0.60, 1.11)	

ATE: Average Treatment Effect; nAbs: anti-MPXV neutralizing antibodies; SFC: Spot-forming colonies ($\times 10^6$ PBMC); *weighted for age and pox vaccination; $ from fitting a standard linear regression model
