# Supplementary material for: Reactogenicity and Immunogenicity Against MPXV of the Intradermal Administration of Modified Vaccinia Ankara Compared to the Standard Subcutaneous Route
Source: Vaccines (Basel). 2024 Dec 31;13(1):32. doi: 10.3390/vaccines13010032 (PMC11769009; doi:10.3390/vaccines13010032)
Supplement: Supplementary file 1 [file vaccines-13-00032-s001.zip › Suppl_Table_S4.pdf]

Supplementary Table S4. Potential average change one month after the completion of vaccination cycle according to the route of administration of the first dose and average treatment effect (ATE) from fitting a marginal model (log_2 scale): analysis restricted to the response to first dose alone in participants primed with smallpox vaccine (N=90, of which 40 through SC route and 50 through ID route).

In PLWH

	Mean (log_2) in ID (95% CI)	Mean (log_2) in SC (95% CI)	ATE* (95% CI)	Interaction p-value$
anti-MPXV IgG	1.87 (1.60, 2.15)	1.55 (1.23, 1.87)	0.32 (–0.10, 0.74)	0.135
anti-MPXV nAbs	1.22 (0.80, 1.64)	1.06 (0.72, 1.39)	0.17 (–0.37, 0.70)	0.536
SFC (Elispot)	0.97 (0.24, 1.71)	0.64 (0.20, 1.09)	0.33 (–0.49, 1.15)	0.431

ATE: Average Treatment Effect; nAbs: anti-MPXV neutralizing antibodies; SFC: Spot-forming colonies ($\times10^6$ PBMC); *weighted for age HIV status
